# Dialysis-Requiring Acute Kidney Injury in Denmark 2000-2012: Time Trends of Incidence and Prevalence of Risk Factors—A Nationwide Study

**DOI:** 10.1371/journal.pone.0148809

**Published:** 2016-02-10

**Authors:** Nicholas Carlson, Kristine Hommel, Jonas Bjerring Olesen, Anne-Merete Soja, Tina Vilsbøll, Anne-Lise Kamper, Christian Torp-Pedersen, Gunnar Gislason

**Affiliations:** 1 Department of Cardiology, Gentofte Hospital, University of Copenhagen, Gentofte, Denmark; 2 Department of Nephrology, Herlev Hospital, University of Copenhagen, Herlev, Denmark; 3 Department of Cardiology, Hvidovre Hospital, University of Copenhagen, Hvidovre, Denmark; 4 Center for Diabetes Research, Gentofte Hospital, University of Copenhagen, Gentofte, Denmark; 5 Department of Nephrology, Rigshospitalet, University of Copenhagen, Copenhagen, Denmark; 6 Institute of Health, Science and Technology, Aalborg University, Aalborg, Denmark; University of Sao Paulo Medical School, BRAZIL

## Abstract

**Introduction:**

Dialysis-requiring acute kidney injury is a severe illness associated with poor prognosis. However, information pertaining to incidence rates and prevalence of risk factors remains limited in spite of increasing focus. We evaluate time trends of incidence rates and changing patterns in prevalence of comorbidities, concurrent medication, and other risk factors in nationwide retrospective cohort study.

**Materials and Methods:**

All patients with dialysis-requiring acute kidney injury were identified between January 1^st^ 2000 and December 31^st^ 2012. By cross-referencing data from national administrative registries, the association of changing patterns in dialysis treatment, comorbidity, concurrent medication and demographics with incidence of dialysis-requiring acute kidney injury was evaluated.

**Results:**

A total of 18,561 adult patients with dialysis-requiring AKI were identified between 2000 and 2012. Crude incidence rate of dialysis-requiring AKI increased from 143 per million (95% confidence interval, 137–144) in 2000 to 366 per million (357–375) in 2006, and remained stable hereafter. Notably, incidence of continuous veno-venous hemodialysis (CRRT) and use of acute renal replacement therapy in elderly >75 years increased substantially from 23 per million (20–26) and 328 per million (300–355) in 2000, to 213 per million (206–220) and 1124 per million (1076–1172) in 2012, respectively. Simultaneously, patient characteristics and demographics shifted towards increased age and comorbidity.

**Conclusions:**

Although growth in crude incidence rate of dialysis-requiring AKI stabilized in 2006, continuous growth in use of CRRT, and acute renal replacement therapy of elderly patients >75 years, was observed. Our results indicate an underlying shift in clinical paradigm, as opposed to unadulterated growth in incidence of dialysis-requiring AKI.

## Introduction

Acute kidney injury (AKI) continues to be a critical illness with profound effects on patient outcomes [1]. Prior epidemiologic studies on AKI have predominantly focused on incidence and outcome measures [2–6], and in spite of reported prevalences >20% amongst hospitalized adults [7], research on comorbid predisposition, and particularly concomitant medication, is sparse. Additionally, identification and management of AKI–and particularly dialysis-requirement–is characterized by regional and national disparities partly due to differing availability of treatment resources; such disparity makes comparison difficult [8,9]. Accordingly, research on “delineating” risk factors for AKI, and development of improved risk stratification, is specifically requested in the recently released *Kidney Disease*: *Improving Global Outcomes* AKI guidelines [10]. Based on data from multiple national registries, we describe the time trends of dialysis-requiring AKI, and the modifiable and non-modifiable risk factors associated with dialysis-requiring AKI, in a national cohort throughout the years 2000 through 2012.

## Materials and Methods

### Data sources

At birth or immigration all Danish residents are issued a unique and permanent civil registration number. Data from national registries is referenced in accordance with the civil registration number, allowing for accurate and effective cross-referencing of national registries. The Danish Health system provides tax-funded health services for all 5½ million Danish citizens.

Information concerning hospital admission and discharge is recorded in the National Patient Registry. Diagnoses and procedures are coded according to the 10th edition of the International Classification of Diseases (ICD)-10, and the Nordic Medico-Statistical Committee Classification of Surgical Procedures, respectively [11–13]. Overall, the National Patient Registry is comprehensive and credible; however, completeness is greatest for primary diagnoses. Additionally, diagnostic and procedural codes are also tied to reimbursement policies, compelling departments to strive for accurate registration, and comorbidities incorporated in the Charlson Comorbidity Index Score have previously been validated with excellent results [14]. Treatment with chronic renal replacement therapy is recorded in the Danish National Registry on Regular Dialysis and Transplantation independent of the National Patient Registry. The registry’s completeness has previously been validated to be >97% [15]. Dispensation of drug prescriptions is recorded in The Danish Register of Medicinal Product Statistics. The registry records information on the Anatomical Therapeutic Chemical Classification System (ATC) code, dispensation date, strength and quantity of all prescriptions dispensed in Denmark [16]. As drug expenses are partially reimbursed the Danish healthcare authorities, all pharmacies are required to provide information to ensure complete and accurate registration [17]. Finally, data on renal function was available in a subpopulation of the study via the Copenhagen General Practitioners Laboratory, which services general practitioners located in the greater Copenhagen area with exception of the municipality of Frederiksberg, corresponding to a population of approximately 1.2 million people. Retrospective register based studies do not need prior ethical approval in Denmark; however, the Danish Data Protection Agency has approved use of data (ref. 2007-58-015 / I-suite nr 00916 GEH-2010-001). All data was anonymized prior to access for this study.

### Study population

**Fig 1:**
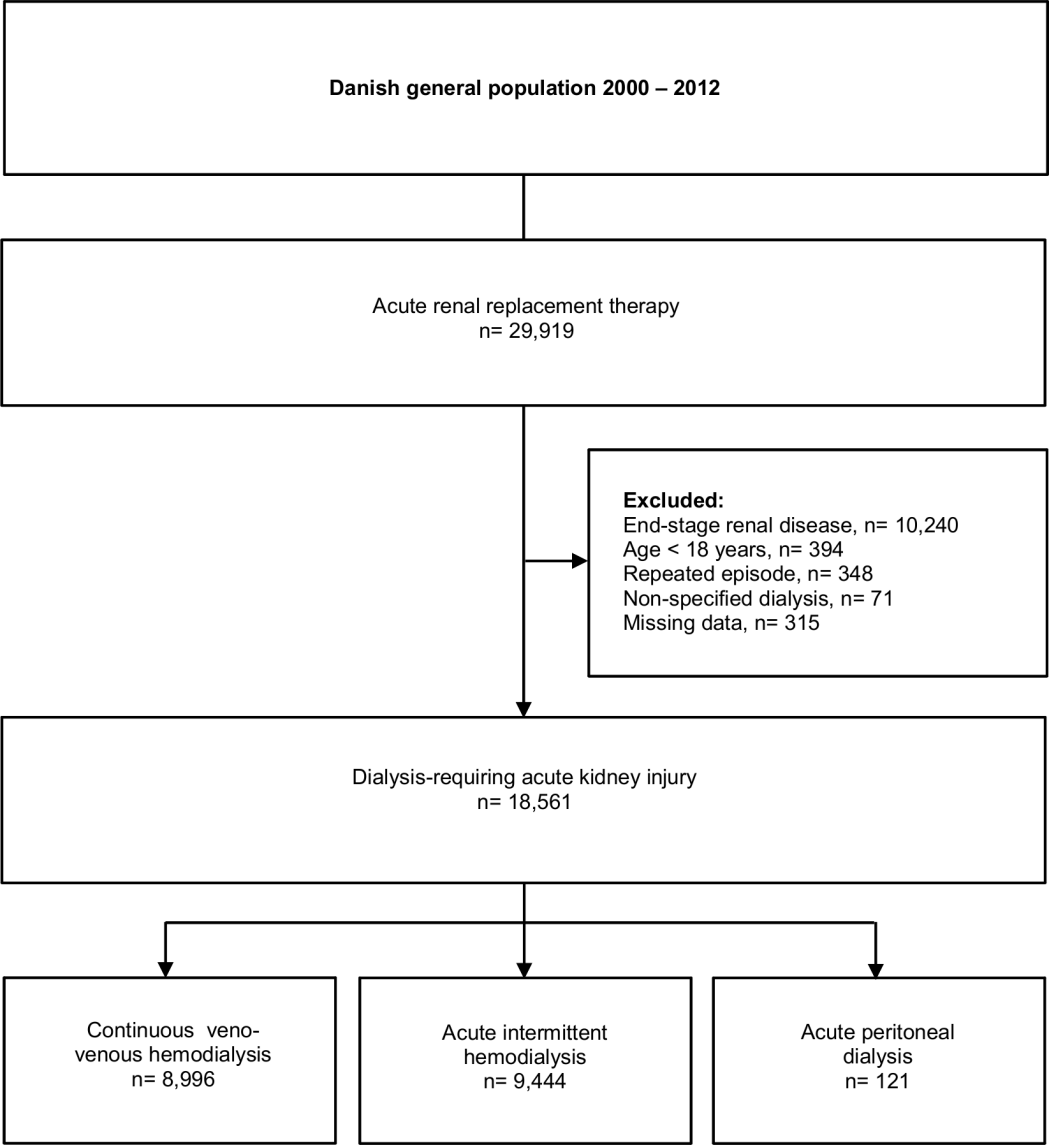
Flow chart depicting study design and exclusion criteria.

Dialysis-requiring AKI was defined as acute dialysis in patients without previously registered end-stage renal disease. All adult patients (≥18 years old) treated with acute dialysis between 1^st^ January 2000 and 31^st^ December 2012 were identified by the acute dialysis procedural code in the National Patient Registry. Patients on chronic renal replacement therapy prior to index were excluded. Verification of AKI by diagnostic code was not prerequisite; ICD-driven validation of AKI in acute dialysis has previously been characterized by inadequate sensitivity [18]. Patients were classified according time period and according to the initial dialysis modality as; acute intermittent hemodialysis, acute peritoneal dialysis, or continuous veno-venous hemodialysis (CRRT). Patients treated with *unspecified* acute dialysis, patients with missing data, and patients previously treated with acute dialysis were excluded. Consequently, only the principal episode was included. A flow chart depicting study design is shown in Fig 1.

### Assessment of comorbidity, medication and hospitalization

Comorbidity was classified according to the Charlson Comorbidity Index [19]. Hypertension, chronic kidney disease (CKD), diabetes, congestive heart failure, cancer, chronic obstructive pulmonary disease, liver disease, and ischemic heart disease were identified by relevant ICD-10 codes in the National Patient Registry. Sensitivities for diabetes, hypertension, and heart failure were augmented by use of data pertaining to prescription medication. The method has previously been described in details [20]. Only comorbidities and prescriptions registered within the last year prior to index were included in the study. Preexisting renal function was assessed via calculation of eGFR using the CKD Epidemiology Collaboration (CKD-EPI) equation amongst a subset of patients (n = 3,131) by averaging all available plasma creatinine measurements 2–52 weeks prior to dialysis-requiring AKI [21]. Admission and discharge dates were identified in the National Patient Registry and the duration of dialysis requirement and hospitalization was calculated. Patients requiring intensive care treatment were identified based on procedural codes denoting respiratory support, circulatory support, or CRRT. Surgical interventions performed ≤14 days prior to index were identified, and classified according to procedural code.

Concurrent outpatient medications were identified based on ATC-code as any prescription redeemed within 90 days prior to index, with exception of non-steroidal anti-inflammatory drugs and antibiotics, where only prescriptions dispensed within 30 days of index were included. Full details of included medications, relevant codes, and algorithms are provided in the supplemental materials (S1 File).

### Statistical analysis

Continuous variables were compared with the Kruskal-Wallis test, and discrete variables with Chi-square test. Population-based incidence rates were calculated from cross-sectional estimates of population size. Trends in crude incidence rates of dialysis-requiring AKI were analyzed using joinpoint regression models. Grid search was applied to determine locations of no more than two joinpoints with subsequent permutation test to determine superiority. Prevalence measures were analyzed using the Mantel-Haenszel test for trend. Durations of dialysis and hospitalization were estimated in a Kaplan-Meier analysis to offeset in-hospital mortality. Fulfillment of model assumptions were tested and found valid unless otherwise stated. Data management and analyses were performed using SAS version 9.4 (SAS Institute Inc.), R version 2.15.2 (R Development Core Team), and the Joinpoint Regression Program, Version 4.0.4 (Statistical Research and Applications Branch, National Cancer Institute). Statistical significance was defined as a two sided p-value <0.0,5, mean results are reported with standard deviation (SD), median results with interquartile range (IQR), and summary results with 95% confidence intervals (CI).

## Results

A total of 18,561 adult patients with dialysis-requiring AKI were identified between January 1^st^ 2000 and December 31^st^ 2012. Patients were predominantly male, >60 years old and burdened with preexisting comorbidity. Baseline characteristics stratified according to time periods are shown in Table 1. Baseline characteristics indexed by initial dialysis modality are shown in Table 2. A total of 6,483 patients were excluded from the study, 6,161 due to preceding end-stage renal disease, and 322 due to recurrent dialysis-requiring AKI.

**Table 1:** Time-stratified baseline characteristics of patients developing dialysis-requiring acute kidney injury in Denmark between 2000 and 2012.

		2000–2003	2004–2008	2009–2012	
		n = 3,457	n = 7,892	n = 7,212	*p-value*
**Characteristics**	Gender (male) % (n)	64.0 (2,221)	63.4 (5,001)	62.4 (4,498)	0.224
	Age (years) mean ±SD	64.1 ±14.1	66.1 ±13.5	66.8 ±13.5	<0.001
	Hospitalization duration (days), median [IQR]	84 [81–87]	81 [79–84]	75 [72–78]	<0.001
	Dialysis duration (days), median [IQR]	22 [20–23]	21 [20–21]	19 [18–19]	<0.001
**Dialysis Modality**	Acute intermittent hemodialysis % (n)	72.2 (2,496)	49.7 (3,923)	41.9 (3,025)	<0.001
	Continuous veno-venous hemodialysis % (n)	27.4 (948)	49.4 (3,894)	57.6 (4,154)	<0.001
	Acute peritoneal dialysis % (n)	0.4 (13)	1.0 (75)	0.5 (33)	0.576
**Comorbidities**	Charlson Index Score (median [IQR]	6 [4–8]	5 [4–8]	5 [4–9]	0.318
	Hypertension % (n)	29.0 (1,002)	33.2 (2,622)	37.0 (2,667)	<0.001
	Congestive Heart Failure % (n)	20.3 (702)	19.1 (1,506)	19.7 (1,418)	0.656
	Diabetes Mellitus % (n)	19.4 (669)	22.3 (1,762)	26.8 (1,933)	<0.001
	Ischemic Heart Disease % (n)	25.2 (871)	20.8 /1,638)	19.1 (1,377)	<0.001
	Chronic Kidney Disease % (n)	33.6 (1,161)	26.6 (2,095)	24.5 (1,770)	<0.001
	Peripheral Vascular Disease % (n)	13.2 (456)	11.6 (918)	10.0 (724)	<0.001
	Cancer % (n)	16.0 (552)	16.9 (1,334)	17.7 (1,273)	0.029
	Liver disease % (n)	8.5 (293)	6.8 (535)	7.8 (559)	0.577
	Chronic pulmonary obstuctive disease % (n)	10.7 (369)	10.6 (837)	11.1 (800)	0.415
**Surgery**	Surgery % (n)	43.2 (1,493)	49.2 (3,885)	47.6 (3,430)	0.002
	Cardiac surgery % (n)	12.1 (417)	12.7 (1,002)	12.7 (913)	0.614
	Gastric surgery % (n)	13.3 (458)	15.6 (1,230)	13.7 (986)	0.004
	Orthopedic surgery % (n)	4.3 (147)	4.9 (388)	5.1 (371)	0.058
**Intensive Care**	Admission to intensive care % (n)	52.9 (1,832)	71.7 (5,657)	75.3 (5,429)	<0.001
	Mechanical ventilation % (n)	39.2 (1,354)	63.8 (5,032)	67.0 (4,833)	<0.001
	Circulatory shock % (n)	15.6 (539)	18.6 (1,468)	26.7 (1,925)	<0.001
**Pharmacotherapy**	No. of singular prescriptions, median [IQR]	2 [1–5]	3 [1–6]	4 [1–6]	<0.001
	Polypharmacy (≥3) % (n)	47.8 (1,651)	56.9 (4,494)	61.2 (4,414)	<0.001
	Polypharmacy (≥5) % (n)	26.0 (897)	36.8 (2,901)	41.0 (2,958)	<0.001

**Table 2:** Modality-stratified baseline characteristics of patients with dialysis-requiring acute kidney injury acute kidney injury in Denmark between 2000 and 2012.

		Continuous veno-venous hemodialysi	Acute intermittent hemodialysis	Acute peritoneal Dialysis	
		n = 8,996	n = 9,444	n = 121	*p-value*
**Characteristics**	Gender (Male), % (n)	63.1 (5,675)	63.1 (5,958)	63.6 (77)	0.992
	Age (years), mean ±SD (n)	65.4 ±13.3	66.6 ±13.9	64.8 ±13.4	<0.001
	Hospitalization duration (days), median [IQR]	84 [82–86]	76 [74–78]	73 [55–82]	<0.001
	Dialysis duration (days), median [IQR]	24 [23–25]	17 [17–18]	24 [12–31]	<0.001
**Comorbidities**	Charlson Index Score, median [IQR]	5 [4–7]	6 [4–9]	5 [4–8]	<0.001
	Hypertension % (n)	31.7 (2,850)	35.9 (3,392)	40.5 (49)	<0.001
	Congestive Heart Failure % (n)	18.9 (1,704)	20.1 (1,894)	23.1 (28)	0.098
	Diabetes Mellitus % (n)	21.4 (1,926)	25.5 (2,411)	22.3 (27)	<0.001
	Ischemic Heart Disease % (n)	22.5 (2,022)	19.4 (1,834)	24.8 (30)	<0.001
	Chronic Kidney Disease % (n)	12.7 (1,141)	40.6 (3,837)	39.7 (48)	<0.001
	Peripheral Vascular Disease % (n)	11.4 (1,021)	11.3 (1,065)	9.9 (12)	0.879
	Cancer % (n)	15.9 (1,433)	18.1 (1,710)	13.2 (16)	<0.001
	Liver disease % (n)	7.5 (677)	7.5 (704)	5.0 (6)	0.564
	Chronic obstructive pulmonary disease % (n)	11.4 (1,023)	10.3 (975)	6.6 (8)	0.024
**Surgery**	Surgery ≤14 days % (n)	61.1 (5,495)	34.5 (3,260)	43.8 (53)	0.001
	Cardiac surgery % (n)	19.1 (1,722)	6.3 (591)	15.7 (19)	<0.001
	Gastric surgery % (n)	19.9 (1,788)	9.2 (867)	15.7 (19)	<0.001
	Orthopedic surgery % (n)	5.1 (460)	4.7 (444)	1.7 (2)	0.110
**Intensive Care**	Admission to intensive care % (n)	100.0 (8996)	40.7 (3,849)	60.3 (73)	<0.001
	Mechanical ventilation % (n)	87.9 (7,908)	34.3 (3,241)	57,9 (70)	<0.001
	Circulatory shock % (n)	30.0 (2,700)	12.9 (1219)	10.7 (13)	<0.001
**Pharmacotherapy**	Number of singular prescriptions (median [IQR]	3 [1–6]	3 [1–6]	4 [1–6]	<0.001
	Polypharmacy (≥3) % (n)	55.2 (4,965)	58.5 (5,525)	57.0 (69)	<0.001
	Polypharmacy (≥5) % (n)	35.6 (3,202)	37.2 (3,510)	36.4 (44)	0.085

## Crude incidence rate

**Fig 2:**
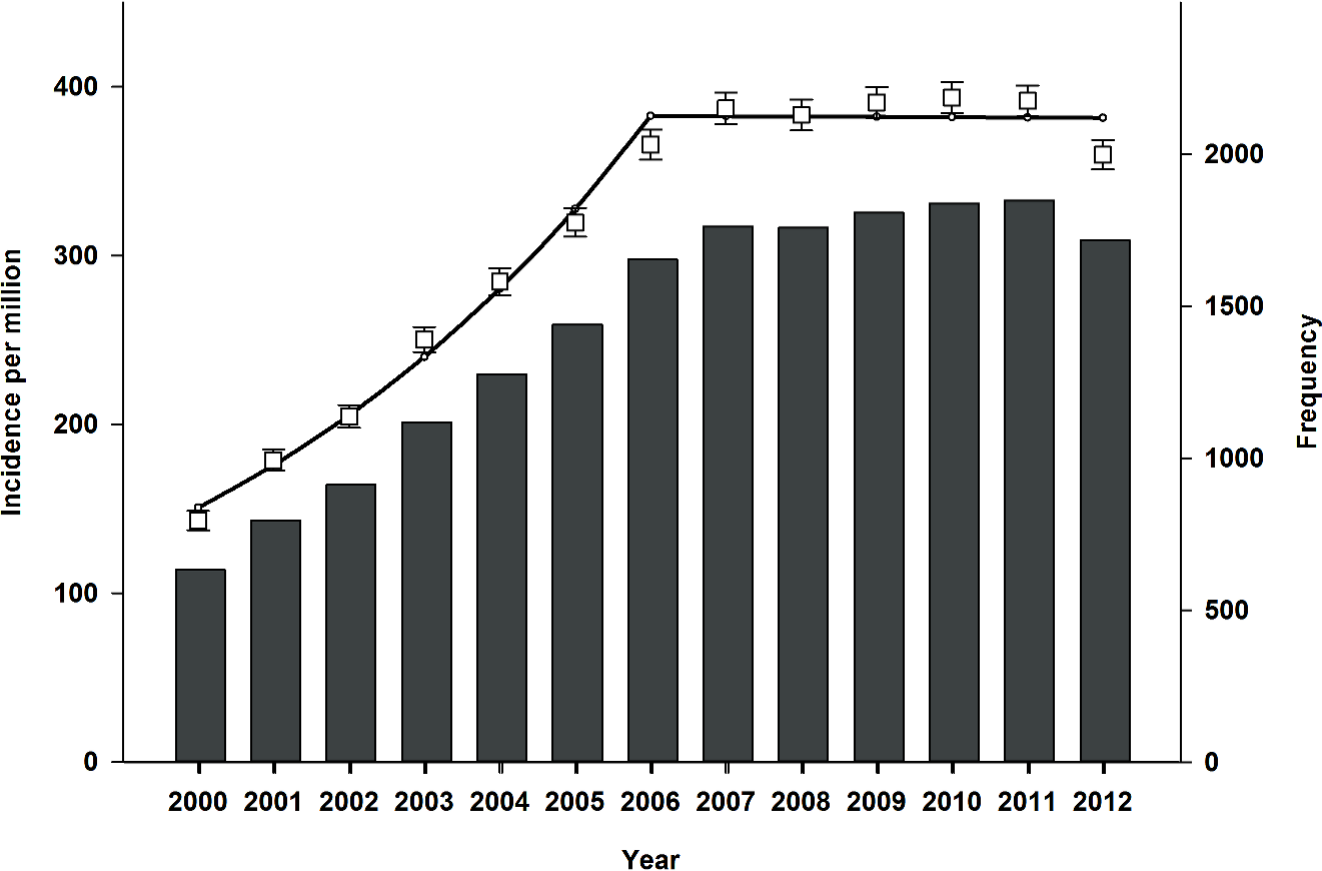
Crude incidence rate per million and frequency of dialysis-requiring acute kidney injury in Denmark between 2000 and 2012. Incidence rate shown as boxplot with 95% CI and regression, and frequency shown as a histogram.

**Fig 3:**
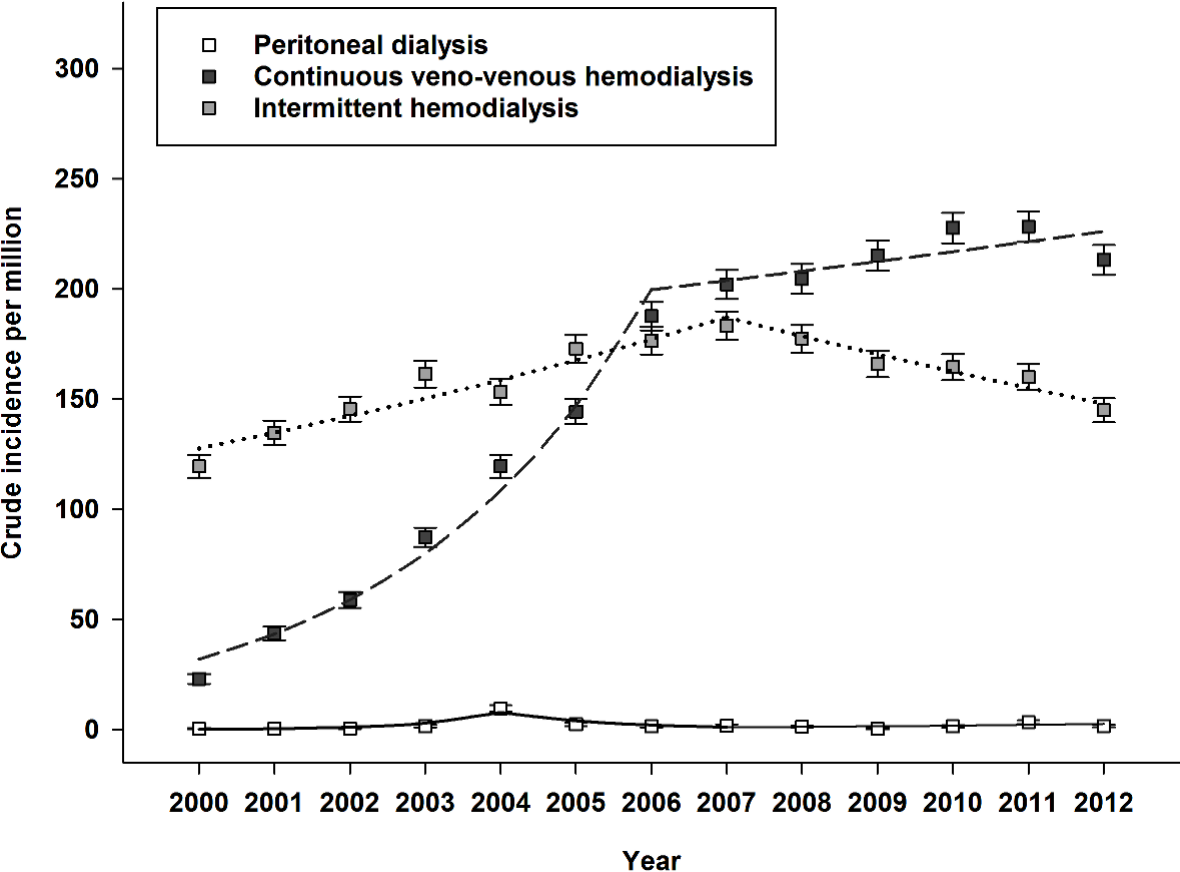
Modality-specific incidence per million of dialysis-requiring acute kidney injury in Denmark between 2000 and 2012. Boxplot with 95% CI and regressions.

**Fig 4:**
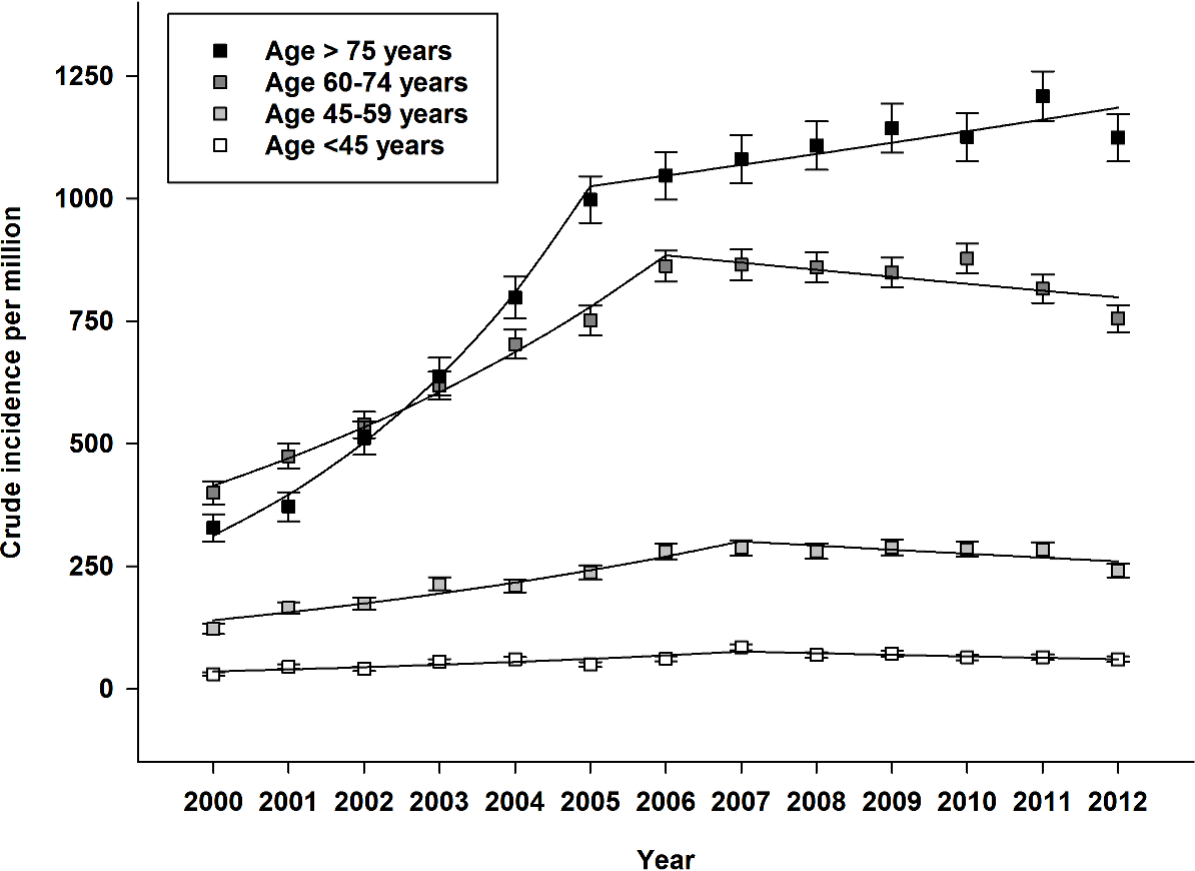
Age-stratified incidence per million of dialysis-requiring acute kidney injury in Denmark between 2000 and 2012. Boxplot with 95% CI and regressions.

**Fig 5:**
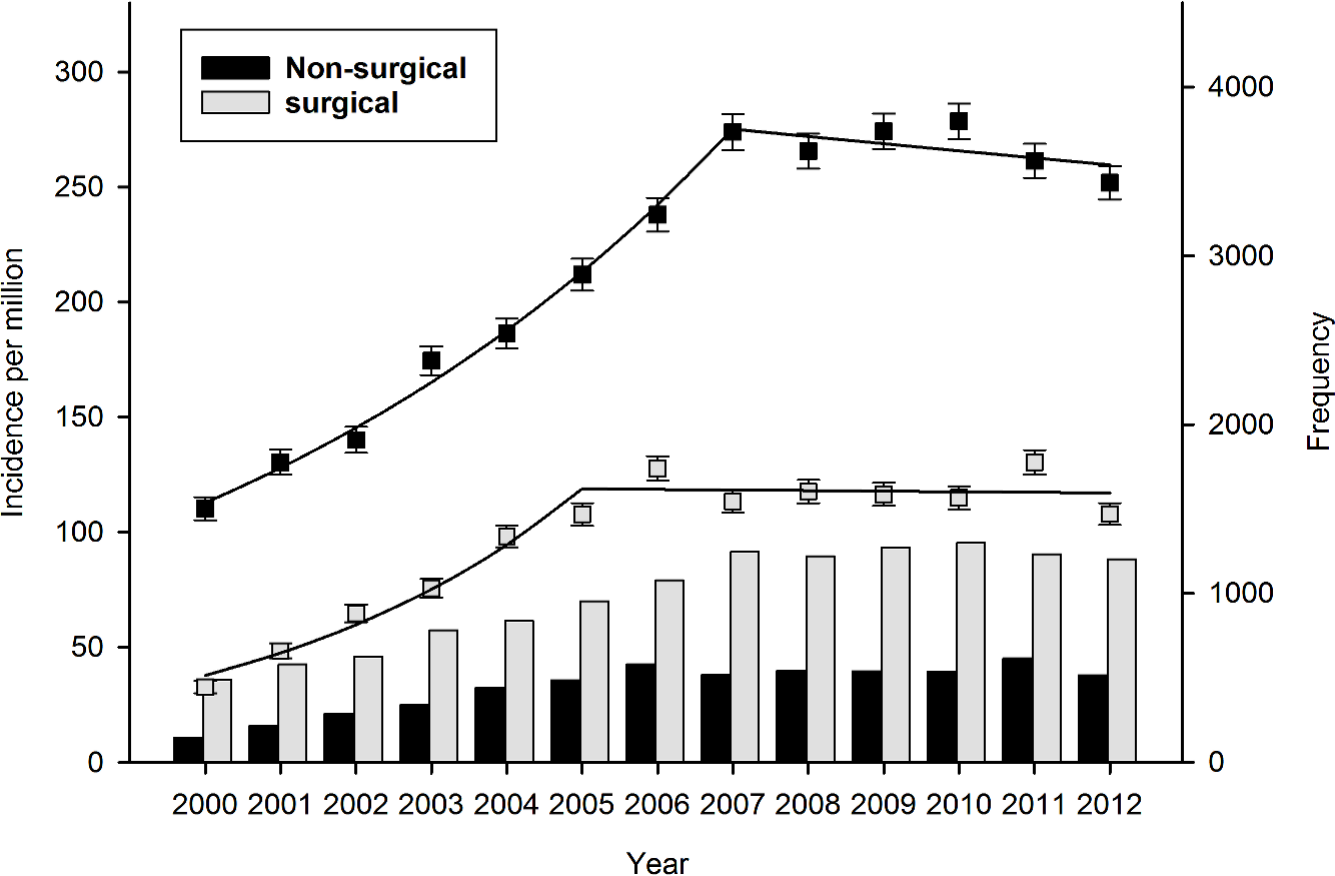
Incidence of surgical and non-surgical dialysis-requiring acute kidney injury per million in Denmark between 2000 and 2012. Incidence rate shown as boxplot with 95% CI and regressions, and frequency shown as a histogram.

Crude incidence rate of dialysis-requiring AKI increased from 143 per million (95%CI 137–144) in 2000 to 360 per million (95%CI 351–368) in 2012. Notably, growth in crude incidence stabilized in 2006. Gender-specific incidence of dialysis-requiring AKI increased from 99 per million (95%CI 92–106) and 186 (95%CI 177–196), to 279 (95%CI 268–289) and 439 (95%CI 425–453), in women and men respectively. Overall crude incidence and annual frequency are shown in Fig 2. Use of CRRT increased continuously throughout the period. Modality-specific crude incidence is shown in Fig 3. Similarly, use of acute renal replacement therapy in elderly patients >75 years also increased throughout the period. Age-stratified incidence of dialysis-requiring AKI is shown in Fig 4. Finally, incidence of both surgical and non-surgical dialysis-requiring AKI stabilized around 2006. Incidence of Dialysis-requiring AKI stratified according to surgical exposure is shown in Fig 5.

### The context of dialysis-requiring AKI

Overall comorbidity adjudged by the Charlson Comorbidity Index Score remained unchanged (p = 0.318). However, the pattern of comorbidity changed substantially over time. Notably, prevalence of hypertension and diabetes increased from 27.7% to 38.9% (p<0.001), and 19.0% to 28.3% (p<0.001), respectively. Conversely, prevalence of preexisting peripheral vascular disease and chronic kidney disease (CKD) decreased from 14.9% to 9.9% (p = 0.021) and 39.2% to 23.3% (p<0.001), respectively. However, modality-specific prevalence of CKD remained stable in patients receiving CRRT (p = 0.081) and acute intermittent hemodialysis (p = 0.547), and prevalence of CKD in acute peritoneal dialysis actually increased (p<0.001). Additionally, the proportion of patients taking ≥3 medications and ≥5 medications increased from 42.8% to 61.5% (p<0.001), and 21.2% to 42.2% (p<0.001), respectively. Notably, the pattern of changes in prevalence of comorbidities in elderly patients >75 years did not differ substantially from the general cohort; prevalence of hypertension and diabetes increased (39.2% to 46.6% (p = 0.002) and 18.4% to 24.4% (p = 0.002), respectively), while prevalence of peripheral vascular disease, ischemic heart disease, congestive heart failure and CKD decreased (17.4% to 12.6% (p = 0.003), 34.5% to 26.3% (p<0.001), 23.1% to 17.8% (p = 0.002) and 44.7% to 32.7% (p<0.001). Finally, the median duration of dialysis and length of hospital stay of elderly patients >75 years decreased from 23 days [IQR 12–45] to 21 days [IQR 11–35] (p = 0.035), and 89 days [IQR 46–120] to 71 days [IQR 40–109].

Patients were admitted due to a multitude of conditions. Although the prevalence of surgical exposure increased from 37.1% to 45.8% (p = 0.002), non-surgical dialysis-requiring AKI remained marginally predominant. Modality-specific proportions were 50.8%, 48.5%, and 0.7%, for acute intermittent hemodialysis, CRRT, and acute peritoneal dialysis, respectively. Only 13.6% of patients were hospitalized due to primary renal disease; other major subgroups included cardiovascular disease (22.9%), gastrointestinal and endocrine disease (16.7%), infectious disease (7.4%) and cancer (3.4%). Notably, the number of patients receiving acute renal replacement therapy in the intensive care unit increased from 52.9% to 75.3% (p<0.001), and dialysis-requiring AKI was increasingly associated with circulatory shock and respiratory failure; the prevalence of circulatory shock and mechanical ventilation increasing from 15.6% to 25.7% (p<0.001) and 39.0% to 67.0% (p<0.001), respectively. Finally, registered eGFRs in patients receiving CRRT, acute intermittent hemodialysis, and acute peritoneal dialysis were 65 ml/min/1.73m^2^ [IQR 47–84], 50 ml/min/1.73m^2^ [IQR 29–72], and 21 ml/min/1.73m^2^ [IQR 13–65], respectively. Comparably, median eGFR increased from 57 ml/min/1.73m^2^ [IQR 35–76] in 2000–2004 to 62 ml/min/1.73m^2^ [IQR 42–84] in 2009–2012 (p<0.001).

## Discussion

This nationwide study encompassing 18,561 patients with severe AKI has several important findings. First, the uninterrupted growth in crude incidence rate of dialysis plateaued at approximately 360 per million from 2006 onwards. Second, the use of CRRT increased substantially throughout the studied period. Notably, an increasing number of patients received acute renal replacement in the intensive care unit in the context of non-renal organ failure. Third, incidence of dialysis-requiring AKI continued to grow throughout the period in elderly patients >75 years.

The rising incidence of dialysis-requiring AKI could reflect a number of reasons. Conceivably, the number of patients developing severe AKI could be increasing, possibly due to demographic change associated with rising longevity. However, the criteria for initiation of dialysis in AKI may also have changed; partly due to increasing capacity, improving prognoses, the introduction of novel dialysis technologies, increasing referral of patients with severe illness, and changing attitudes amongst the general population with regard to treatment expectancy. Yet few studies have previously addressed the incidence rate of dialysis-requiring AKI in general populations. Published incidence rates suggest persistent increase with recent annual growth >10% [2,6,22–25]. However, updated and comprehensive information is limited, and major publications have predominantly been based on data from the US.

Overall, an increase in dialysis-requiring AKI in accord with previously published estimates was observed [23,25]. However, annual growth stagnated and incidence rate remained fixed from 2006 onwards. A number of considerations are pertinent: First, the observed incidence rates in our study, albeit not incompatible with previous observations, are of smaller magnitude. Arguably, as previous incidence rates were estimated from subpopulation sampling, and calculated using an algorithm with sensitivity issues regarding the discrimination between dialysis-requiring AKI and acute dialysis in end-stage renal disease [26], sampling bias could be an issue; particularly, as recurrent admissions with AKI has been observed to characterize the trajectory towards end-stage renal disease [27]. Our results employ a modified algorithm for identification of dialysis-requiring AKI; as such certain disparities are to be expected. Additionally, incidence of AKI in the US has previously–and recently–been reported to be greater than in northern Europe [7]; thus, the observed difference could represent genuine geographical variation. Second, incidence of dialysis-requiring AKI was observed to be dependent on age, and growth in incidence rate was greatest amongst elderly patients >75 years. Additionally, growth and stagnation in incidence of AKI coincided with absolute and proportional increase in use of CRRT. The introduction of CRRT into common clinical practice did not occur until the end of the 1990’s [28]. Treatment of dialysis-requiring AKI in patients admitted to intensive care units is commonly the shared responsibility of intensive care specialists and consulting nephrologists. However, following the introduction CRRT, initiation of acute renal replacement therapy in critical illness has shifted from the nephrology departments to intensive care units. Consequently, the observed growth in incidence of dialysis-requiring AKI could plausibly reflect expanding use of acute renal replacement therapy in novel populations, coupled with the changing economic priorities in Danish health care throughout the last decade, as opposed to genuine growth in incidence of severe AKI. Finally, a number of countermeasures; including pre-emptive saline hydration prior to contrast exposure, discontinuation of renin-angiotensin system-blocking agents and metformin in acute illness, and early goal-directed therapy as suggested in the *surviving sepsis* guidelines [29], were introduced throughout the 2000’s. Although uncertain, the attenuated growth in incidence could also represent the combined effect of these interventions.

Various modifiable and non-modifiable risk factors are associated with AKI [30–33]. We observed a male predominance, an increased propensity for use of acute renal replacement amongst elderly, and an overall increase in comorbidity. First, although gender is known not to infer increased risk of non-dialysis-requiring AKI [4,34], the significance of gender on risk of dialysis-requiring AKI remains contested [35,36]. Comparably, end-stage renal disease is more common amongst men, despite no difference in prevalence of CKD [37,38], and recent publications have repeatedly found male gender to be associated with increased risk of dialysis-requiring AKI [6,39]. As such, the predominance of male gender observed in our population may plausibly reflect a gender-specific inherent increased risk for severe renal injury. Second, the context of dialysis-requiring AKI seems to have changed over time. Plausibly due to the influx in use of CRRT, dialysis-requiring AKI was increasingly associated with non-renal organ failure. Throughout the last decade an ever greater proportion of patients with dialysis-requiring AKI initiate treatment with CRRT in the intensive care unit [24,40]. Possibly due the increasing burden of circulatory failure [41,42], and the increase in concurrent respiratory failure [6,24,42,43]. Nonetheless, our results reiterate a trend towards greater prevalence of intensive care requirement. Additionally, contradictory prevalence measures of different comorbidities were observed. Although prevalence of hypertension and diabetes increased, prevalence of peripheral vascular disease and ischemic heart disease decrease. However, although not unambiguous, similar patterns have been observed in other dialysis-requiring AKI populations [24,40]. As such, the observed growth and stabilization of incident dialysis-requiring AKI could be representative of a general change in clinical paradigm, rather than genuine growth in dialysis-requiring AKI. Furthermore, considering how incidences of non-dialysis and dialysis-requiring AKI following cardiac surgery have increased throughout the last decade [43], and how surgery, particularly cardiovascular and gastric surgery, contributes to ⅓ of all AKIs [44], the increase in use of acute renal replacement therapy is perhaps not surprising. Additionally, although geriatric surgery is increasingly common, surgical mortality amongst elderly patients is decreasing [45]. With outcomes for elderly surgical patients improving, the observed increase in prevalence of surgery amongst patients with dialysis-requiring AKI does not plausibly seem to be wholly attributable to mere demographic shift. As such, the observed changing growth patterns in incidence of dialysis-requiring AKI could in part represent the impact of improving survival outcomes in previously marginalized patients, and in part the impact of changing thresholds for initiation of acute renal replacement therapy.

Our study had several major strengths. First, consequent to the structure of health care in Denmark, national registries record information, without exception, on every hospitalization and hospital-based treatment provided to Danish citizens; the strength of the registries ensures abundant and almost faultless follow-up. Additionally, the national registries are extensively validated with excellent results, and consequently, demographic sampling bias is minimized. Second, accurate dissemination between dialysis-requiring AKI and acute dialysis in end-stage renal disease was ensured due to the availability of reliable data from the Danish National Registry on Regular Dialysis and Transplantation. Nevertheless, identification of genuine dialysis-requiring AKI in registry-based research remains a challenge. Our algorithm does not require registration of a billing code denoting AKI; consequently, our algorithm does not consider alternate indications for acute renal replacement therapy to AKI. However, application of acute renal replacement therapy for such treatment remains exceptional, and the effects on overall incidence rates plausibly negligible. Additionally, the benefits of including an AKI-diagnosis in our algorithm remain debatable. In Denmark, procedure-performing departments are reimbursed for their work according to procedure coding. Therefore accurate and complete dialysis procedure coding is of immense financial importance for nephrology departments. Simply put, departments are reimbursed per registered dialysis session. Additionally, the accuracy of CRRT procedure coding has previously been validated; the positive predicative value was 98% [46]. Conversely, diagnostic coding is only partially tied to reimbursement; only the principal diagnostic code is used to determine reimbursement. As such, the addition of an AKI-diagnostic code to the algorithm, although possibly associated with improved specificity and positive predicative value, would likely increase propensity for ‘code creep’ and decrease sensitivity and positive predicative values.

Finally a number of specific limitations apply to our results: First, prevalence of comorbidities may be underestimated due to reliance on diagnosis codes registered in-hospital, and the registries employed do not catalog body-mass-index, alcohol- and tobacco consumption or proteinuria; thus, a number of important risk factors remain unaddressed. Second, due to the registry-based nature of the study, results remain at risk of confounding due to issues related to administrative coding and changes in reimbursement policies. Third, calculation of eGFRs was only possible in patients with available serum creatinine measurement. As such, the eGFRs may be subject to detection and demographic biases. Fourth, as the ethnic composition of Denmark is predominantly northern European and relatively homogenous, extrapolation of results to other ethnic groups would not necessarily be appropriate, and finally, due to the inherent nature of epidemiological data, definite causal extrapolation cannot alone be based on the presented results.

## Conclusions

Crude incidence rate of dialysis-requiring AKI in Denmark stabilized from 2006 onwards. Notably, growth and stagnation coincided with changing patterns in incidence of CRRT and use of acute renal replacement therapy in elderly. Thus, the previously observed persistent increase in crude incidence rate of dialysis-requiring AKI was not confirmed. As dialysis-requiring AKI was increasingly associated with non-renal organ failure, increased comorbidity and older age, our results provide evidence of a shift in clinical paradigm.

## Supporting Information

S1 File Supplemental Methods. (DOCX)

## Abbreviations

CI: Confidence Interval
AKI: Acute Kidney Injury
ATC: Anatomical Therapeutic Chemical Classification System
CKD: Chronic Kidney Disease
CRRT: Continuous Veno-Venous Hemodialysis
eGFR: Estimated Glomerular Filtration Rate
ICD: International Classification of Diseases
IQR: Interquartile Range
SD: Standard Deviation
