# Supplementary material for: Dialysis-Requiring Acute Kidney Injury in Denmark 2000-2012: Time Trends of Incidence and Prevalence of Risk Factors—A Nationwide Study
Source: PLoS One. 2016 Feb 10;11(2):e0148809. doi: 10.1371/journal.pone.0148809 (PMC4749171; doi:10.1371/journal.pone.0148809)
Supplement: S1 File — (DOCX) [file pone.0148809.s001.docx]

**Supplemental Methods**

Dialysis modalities were identified based on the Nordic Medico-Statistical Committee Classification of Surgical Procedures as; acute intermittent hemodialysis (procedural code ‘BJFD00’), acute peritoneal dialysis (procedural code ‘BJFD01’), continuous renal replacement therapy (procedural code ‘BJFD02’) or *unspecified* acute dialysis (procedural code ‘BJFD0’).

Diabetes was identified by diagnosis with ICD-10 code ‘DE10-14’ or by prescription of any anti-diabetic medication defined as ATC code ‘A10’. Classification as type 1 diabetes was based on ICD-10 code or insulin mono-therapy established before 40 years of age. Hypertension was identified by ICD-10 code ‘DI1’ or by prescription of any combination of 2 concomitant antihypertensive medications defined as ATC codes ‘C02’, ‘C03’, ‘C07’, ‘C08’ and ‘C09’. Congestive heart failure was identified by ICD-10 code ‘DI50’. Anti-congestive treatment was defined by ATC codes as ’C07’ (β-blockers), ‘C09’ (RAS blocking agents), ‘C03CA’ (Loop-diuretics) and ‘C03DA’ (Aldosterone Antagonists). CKD was identified by ICD-10 codes ‘DN18’ (chronic renal insufficiency), ‘DN19’ (renal insufficiency), ‘DN391’ (chronic proteinuria), ‘DN25-DN29’ (other disorders of the kidney and ureter), ‘DN03-DN08’ (glomerular diseases) ‘DN11’ (Chronic tubulo-interstitial nephritis), ‘DN14’ (Drug- and heavy-metal-induced tubulo-interstitial and tubular conditions), ‘DE102’ and ‘DE112’ (Diabetic nephropathy), ‘DI12-13’ (hypertensive nephropathy), ‘DQ61’ (cystic kidney disease) and ‘DN06’ (proteinuria). Finally, ischemic heart disease was identified by ICD-10 codes ‘DI20-25’.

Admission length was calculated as the time between first admission and last discharge. The duration of hospitalization was calculated at the time between first admission and last discharge. The duration of dialysis-requiring AKI was calculated as the time between first and last acute dialysis treatment. Information regarding admission to an intensive care unit was defined by treatment with respiratory support (procedural code ‘BGD’), circulatory support (procedural code ‘BFHC92’) or CRRT (procedural code ‘BJFD02’) based on records in the National Patient Registry. Correspondingly, surgical status was defined on the basis of surgical procedures performed within 14 days prior to index. Surgery was classified as cardiac (procedural code ‘KF’), gastric (procedural code ‘KJC’ to ’KJN’), orthopedic (procedural code ‘KN’), endoscopic (procedural code ‘KU’), or other (all remaining procedural codes except specific codes related to placement of peritoneal catheters).

Finally, the number of singular prescriptions was calculated based on treatment with any medication amongst the following ATC-codes; ‘A10BA’ – ‘A10BX’ (anti-diabetics), ‘C02A’ – ‘C09X’ (antihypertensives and diuretics), ‘C10A’ – ‘C10B’ (statins), ‘L’ (chemotherapeutics), ‘A02B’ (proton pump inhibitors), ‘B01AC’ (antithrombotic agents), ‘J01’ (antibiotics), ‘J02’ (antimycotics), and ‘M01A’ – ‘M01B’ (NSAIDs).
